# Dislocation of the gastric conduit reconstructed via the posterior mediastinal route is a significant risk factor for anastomotic disorder after McKeown esophagectomy

**DOI:** 10.1002/ags3.12496

**Published:** 2021-08-12

**Authors:** Masanobu Nakajima, Hiroto Muroi, Maiko Kikuchi, Junki Fujita, Keisuke Ihara, Masatoshi Nakagawa, Shinji Morita, Takatoshi Nakamura, Satoru Yamaguchi, Kazuyuki Kojima

**Affiliations:** ^1^ First Department of Surgery Dokkyo Medical University Shimotsugagun Japan; ^2^ Department of Surgery Dokkyo Medical University Nikko Medical Center Nikko Japan

**Keywords:** anastomotic leak, esophageal neoplasms, esophagectomy, morbidity, quality of life

## Abstract

**Background:**

Anastomotic disorder of the reconstructed gastric conduit is a life‐threating morbidity after thoracic esophagectomy. Although there are various reasons for anastomotic disorder, the present study focused on dislocation of the gastric conduit (DGC).

**Methods:**

The study cohort comprised 149 patients who underwent transthoracic esophagectomy. The relationships between DGC and peri‐ and postoperative morbidities were analyzed retrospectively. Data were analyzed to determine whether body mass index (BMI) and extension of the gastric conduit were related to DGC. Uni‐ and multivariate Cox regression analyses were performed to identify the factors associated with anastomotic disorder.

**Results:**

DGC was significantly related to anastomotic leakage (*P* < .001), anastomotic stricture (*P* = .018), and mediastinal abscess/empyema (*P* = .031). Compared with the DGC‐negative group, the DGC‐positive group had a significantly larger mean preoperative BMI (23.01 ± 3.26 kg/m^2^ vs. 21.22 ± 3.13 kg/m^2^, *P* = .001) and mean maximum cross‐sectional area of the gastric conduit (1024.75 ± 550.43 mm^2^ vs. 619.46 ± 263.70 mm^2^, *P* < .001). Multivariate analysis revealed that DGC was an independent risk factor for anastomotic leakage (odds ratio: 4.840, 95% confidence interval: 1.770‐13.30, *P* < .001). Body weight recovery tended to be better in the DGC‐negative group than in the DGC‐positive group, although this intergroup difference was not significant.

**Conclusion:**

DGC reconstructed via the posterior mediastinal route is a significant cause of critical morbidities related to anastomosis. In particular, care is required when performing gastric conduit reconstruction via the posterior mediastinal route in patients with a high BMI.

## Graphical abstract

Anastomotic disorder is a life‐threating morbidity after esophagectomy. Anastomotic leakage, stricture, and mediastinal abscess/empyema are significantly related to dislocation of the gastric conduit.

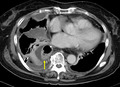

## 1 INTRODUCTION

Esophageal cancer is the seventh most common type of cancer and the sixth leading cause of overall mortality worldwide.^1^ Despite recent developments in surgical technique and perioperative management, esophagectomy for esophageal cancer is one of the most invasive gastroenterological surgeries with a very high morbidity rate.^2^, ^3^, ^4^, ^5^ Anastomotic disorder of the reconstructed organs, especially the gastric conduit, is a common, severe, and life‐threatening complication of esophagectomy.^6^, ^7^

Esophageal reconstruction with a gastric conduit is typically performed via the subcutaneous, retrosternal, or posterior mediastinal (PM) route.^8^, ^9^, ^10^ Among these three routes, the PM route is considered the most physiological.^11^, ^12^ However, PM reconstruction carries a risk of severe morbidities such as mediastinal abscess, empyema, and tracheobronchial fistula.^13^, ^14^, ^15^, ^16^ Therefore, it is important to identify the risk factors for anastomotic disorder of the gastric conduit reconstructed through the PM route.

Previous studies have reported that anastomotic disorder, especially anastomotic leakage, results from ischemia of the gastric conduit,^17^, ^18^ compression^19^ or tension^20^, ^21^ of the anastomotic site, or poor anastomotic technique.^22^ However, dislocation of the gastric conduit (DGC) is rarely discussed.

DGC is often seen after esophagectomy and has an unfavorable effect on postoperative quality of life (QOL) in patients who have undergone esophagectomy in clinical practice. However, this seems to be an empirical finding. Despite a thorough search, we could not find any previous reports on the relationship between esophagectomy and DGC.

In this study, we investigated the risk of post‐esophagectomy anastomotic disorder of the gastric conduit reconstructed through the PM route, focusing on DGC.

## 2 METHODS

### 2.1 Patients

We retrospectively reviewed the medical records of 273 patients with esophageal cancer who underwent radical thoracic esophagectomy and gastric conduit reconstruction between January 2009 and December 2018 at Dokkyo Medical University Hospital. We excluded patients who underwent transhiatal esophagectomy (28 cases) and salvage esophagectomy after definitive chemoradiotherapy (nine cases) or laryngopharyngoesophagectomy (four cases). We also excluded 69 cases of retrosternal reconstruction and one of subcutaneous reconstruction. Thus, data from 163 patients who underwent McKeown esophagectomy with gastric conduit reconstruction via the PM route were extracted. Of these 163 patients, we excluded nine who underwent hand‐sewn anastomosis and five who underwent mechanical anastomosis using a linear stapler because we performed these anastomoses on rare occasions in unfavorable condition, such as in patients with a short gastric conduit or short remnant esophagus.

The final study cohort comprised 149 patients who underwent thoracoscopic or transthoracic subtotal esophagectomy and open or hand‐assisted laparoscopic esophageal reconstruction with a gastric conduit via the PM route and cervical stapled anastomosis using a circular stapler.

The patients were staged using the TNM classification (8th edition) of the American Joint Committee on Cancer and the International Union Against Cancer. Informed consent for surgery was obtained from all patients in accordance with our institutional guidelines. The study was approved by the Medical Ethics Committee of Dokkyo Medical University (approval number: R‐20‐7J).

### 2.2 Esophageal reconstruction

After subtotal esophagectomy, abdominal lymphadenectomy and gastric conduit reconstruction was started with the patient in the supine position. The open method was used in 122 cases, while hand‐assisted laparoscopic surgery (HALS) was performed in 27 cases. Gastrectomy was performed to remove the mobilized esophagus, with lymphadenectomy around the left gastric artery and celiac artery. After mobilization of the full stomach and esophagus, a gastric conduit with a width of 3.5‐4 cm was created by dividing the lesser curvature of the stomach. The right gastric artery and right gastroepiploic artery provided the vascular supply to the created gastric conduit. The gastric conduit was pulled up via the PM route. The esophagogastrostomy was performed in the neck by end‐to‐side stapled anastomosis. A 21‐mm or 25‐mm intraluminal circular stapler was used as the stapling device (CDH21, CDH25, Ethicon Ltd.; EEA21, EEA25, Medtronic). The inserted part of the gastric conduit was closed using a linear stapling device (ECHELON FLEX 60, Powered ECHELON FLEX 60, ECR60D, Ethicon Ltd.; Endo GIA tristaple, EGIA60AMT, Medtronic).

### 2.3 Definition of DGC

**FIGURE 1:**
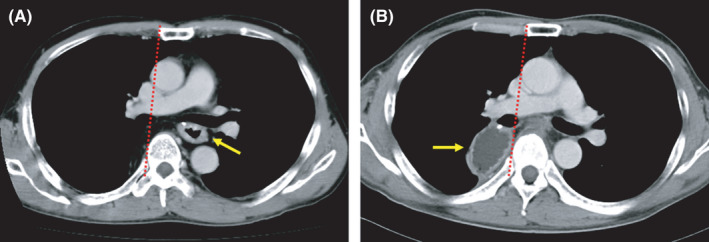
Computed tomography images of the gastric conduit. (A) DGC‐negative case. The gastric conduit (arrow) is in the usual posterior mediastinal space. (B) DGC‐positive case. The gastric conduit (arrow) is located outside of the line on the right edge of the sternum and thoracic vertebral body (dotted line) on the axial view. DGC, dislocation of the gastric conduit

We focused on DGC as a risk factor for anastomotic leakage because DGC results in tension at the anastomotic site. In cases where the gastric conduit is reconstructed via the PM route, the gastric conduit usually dislocates to the right pleural cavity because the mediastinal pleura is resected with the thoracic esophagus. DGC was defined as dislocation of more than 2/3 of the width of the gastric conduit to the right side from the line on the edge of the sternum and thoracic vertebral body (Figure 1).

Imaging assessment

DGC was assessed on the axial view of the first computed tomography (CT) scan. If there were no symptoms of postoperative morbidity, we routinely performed CT 3 months after esophagectomy. When postoperative morbidity was suspected, we immediately performed CT. The earliest CT among this series was performed on postoperative day 1. The maximum cross‐sectional area of the gastric conduit was measured by setting the region of interest with Nazca View ver. 3.2.10000.20 (AstroStage Inc).

### 2.4 Definitions of peri‐ and postoperative morbidities

Peri‐ and postoperative morbidities were defined as complications that seemed to be related to the reconstruction procedure. Complications were assessed in accordance with the Clavien‐Dindo classification, and complications of grade II or above were regarded as significant.

### 2.5 Comparison between preoperative body mass index and DGC

Patients with a high body mass index (BMI) may have a small PM space after esophagectomy and a large volume of greater omentum associated with the gastric conduit. As a result, DGC may occur more easily in patients with a high BMI than in patients with a normal BMI. Therefore, we compared the preoperative BMI between the DGC‐positive and DGC‐negative groups.

### 2.6 Examination of the relationship between extension and DGC

We noticed that the gastric conduits that had dislocated to the pleural cavity tended to be more extended than the non‐dislocated gastric conduits. Therefore, we examined the relationship between the maximum cross‐sectional area of the gastric conduit and DGC.

### 2.7 Examination of body weight change

Body weight change was examined as an indicator of long‐term QOL.

### 2.8 Statistical analysis

The chi‐squared test and Fisher's exact test were used for statistical comparisons of nominal variables where appropriate. Continuous data were compared with the Student's *t*‐test or analyzed by repeated measures one‐way analysis of variance. Multivariate analysis was performed using logistic regression. Differences were considered significant if the *P* value was ˂.05. All statistical analyses were carried out using EZR (version 1.54) (Saitama Medical Center, Jichi Medical University), which is a graphical user interface for R (The R Foundation for Statistical Computing).

## 3 RESULTS

### 3.1 Patients' characteristics and DGC

The characteristics of the eligible patients are described in Table 1. In summary, the population was typical for Japanese patients with esophageal cancer. The mean age was 65.15 years, and the main histologic type was squamous cell carcinoma (91.9%). Sixty‐seven cases (45.0%) were node‐positive clinically, and 35 patients (23.5%) received neoadjuvant chemotherapy using the cisplatin and 5‐fluorouracil regimen or the docetaxel, cisplatin, and 5‐fluorouracil regimen. Thoracoscopic esophagectomy was performed in 77 patients (51.7%), while HALS was performed in 27 patients (18.1%). The mean BMI was 21.91 kg/m^2^. DGC was detected in 57 patients (38.3%). Although we did not decide on the level of measurement, the maximally dislocated level was just below the bifurcation in almost all patients.

**TABLE 1:** Characteristics of the 149 eligible patients who underwent esophagectomy

Variable	Value
Age in years, mean ± SD	65.15 ± 8.45
Sex (male/female)	120/29
Tumor location (upper/middle/lower)	17/79/53
Histology (SCC/AC/other)	132/10/7
T (1/2/3/4)	60/18/65/6
N (0/1/2/3)	82/31/20/16
M (0/1)	142/7
Stage (I/II/III/IVA/IVB)	53/35/46/8/7
NAC (yes/no)	35/114
Preoperative BMI in kg/m^2^, mean ± SD	21.91 ± 3.29
TTE/TSE	72/77
Laparotomy/HALS	122/27
Operation time in minutes, mean ± SD	461.41 ± 73.24
Blood loss in g, median [range]	350 [30‐2090]
Circular stapler size (21 mm/25 mm)	19/130
DGC (yes/no)	57/92

Abbreviations: AC, adenocarcinoma; BMI, body mass index; DGC, distension of the gastric conduit; HALS, hand‐assisted laparoscopic surgery; M, distant metastasis; N, lymph node metastasis; NAC, neoadjuvant chemotherapy; SCC, squamous cell carcinoma; SD, standard deviation; T, depth of tumor invasion; TSE, thoracoscopic esophagectomy; TTE, transthoracic open esophagectomy.

### 3.2 DGC and peri‐ and postoperative morbidities

**FIGURE 2:**
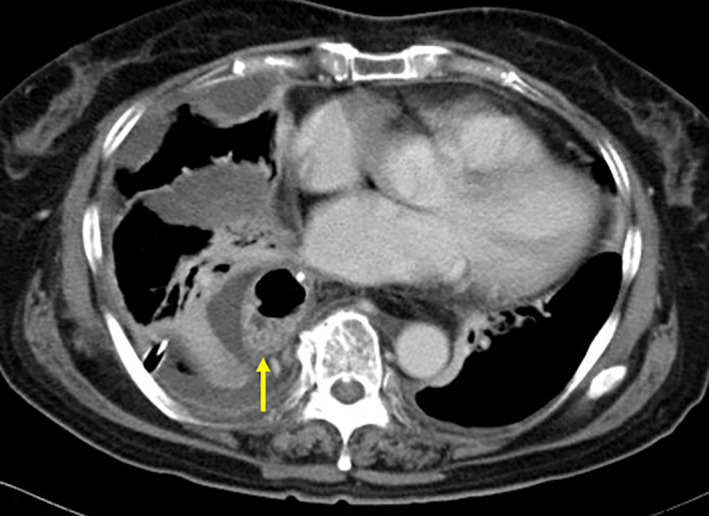
A case of empyema. The dislocated gastric conduit (arrow) with pleural fluid collection and pulmonary atelectasis is seen in the right pleural cavity

Compared with the DGC‐negative group, the DGC‐positive group had significantly higher incidences of anastomotic leakage (*P* < .001) and anastomotic stricture (*P* = .018). Mediastinal abscess and empyema occurred in six patients, five (83.3%) of whom were in the DGC‐positive group (*P* = .031) (Figure 2). Among the 29 cases of stricture, 12 cases (41.4%) had anastomotic leakage. Meanwhile, five patients (83.3%) had anastomotic leakage among six patients with mediastinal abscess and empyema. The incidence of pneumonia, which may be caused by pulmonary atelectasis due to DGC, was not increased in the DGC‐positive group. Delayed gastric emptying (DGE) is a typical postoperative disorder of esophagectomy. In this study, DGE tended to be more frequent in the DGC‐positive group although the increase in frequency was not significant (*P* = .070; Table 2).

**TABLE 2:** Relationship between dislocation of the gastric conduit and anastomosis‐related postoperative morbidities

Morbidity	DGC		*P* value
	Negative	Positive	
Anastomotic leakage			
No	85	39	<.001
Yes	7	18	
Anastomotic stricture			
No	80	40	.018
Yes	12	17	
Mediastinal abscess/empyema			
No	91	52	.031
Yes	1	5	
Pneumonia			
No	86	51	.537
Yes	6	6	
Delayed gastric emptying			
No	81	43	.070
Yes	11	14	

Abbreviation: DGC, distension of the gastric conduit.

### 3.3 Preoperative BMI and DGC

**FIGURE 3:**
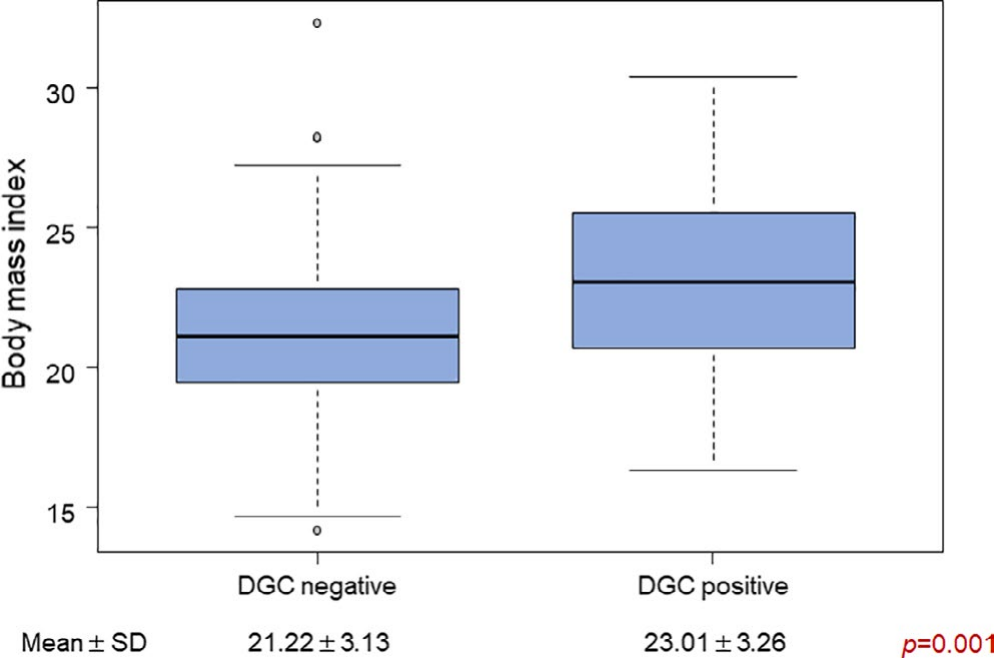
Relationship between DGC and BMI. The BMI of the DGC‐positive group is significantly larger than that of the DGC‐negative group. BMI, body mass index; DGC, dislocation of the gastric conduit; SD, standard deviation

The mean preoperative BMI in the DGC‐negative group (21.22 ± 3.13 kg/m^2^) was significantly smaller than that in the DGC‐positive group (23.01 ± 3.26 kg/m^2^, *P* = .00108; Figure 3).

### 3.4 Relationship between extension and DGC

**FIGURE 4:**
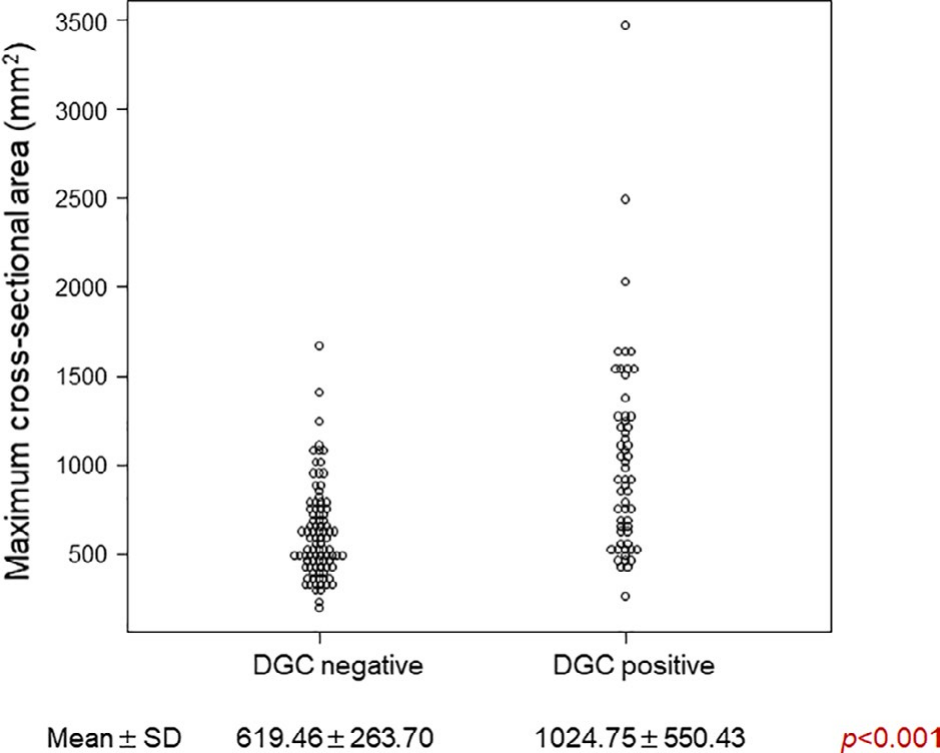
Relationship between dislocation and extension of the gastric conduit. The maximum cross‐sectional area is significantly larger in DGC‐positive patients than in DGC‐negative patients. DGC, dislocation of the gastric conduit; SD, standard deviation

The mean maximum cross‐sectional area of the gastric conduit was significantly smaller in the DGC‐negative group (619.46 ± 263.70 mm^2^) than in the DGC‐positive group (1024.75 ± 550.43 mm^2^, *P* < .001) (Figure 4).

### 3.5 Factors associated with anastomotic disorder

Univariate Cox regression analysis revealed that the factors significantly associated with anastomotic leakage were the preoperative BMI, thoracoscopic esophagectomy, HALS, and DGC (all *P* < .05). These factors were entered in the multivariate model. Multivariate analysis revealed that DGC was an independent risk factor for anastomotic leakage (odds ratio: 4.840, 95% confidence interval: 1.770‐13.30, *P* < .001) (Table 3). Uni‐ and multivariate analyses were also performed to identify the factors associated with anastomotic stricture, mediastinal abscess/empyema, and entire anastomotic disorders. However, no independent risk factors were identified (data not shown).

**TABLE 3:** Cox regression analysis to identify the factors associated with anastomotic leakage

Factors	Univariate analysis			Multivariate analysis		
	OR	95% CI	*P* value	OR	95% CI	*P* value
Age						
<65 years/≥65 years	1.120	0.473‐2.640	.798			
Sex						
Female/male	3.200	0.710‐14.40	.130			
Tumor location						
Middle or lower/upper	1.630	0.483‐5.480	.432			
Stage						
I–II/III–IV	0.396	0.148‐1.060	.065			
NAC						
No/yes	0.285	0.063‐1.280	.102			
Preoperative BMI						
<21.9 kg/m^2^/≥21.9 kg/m^2^	4.240	1.590‐11.40	.004	2.290	0.774‐6.750	.134
TSE						
No/yes	3.600	1.350‐9.630	.011	2.750	0.942‐8.000	.064
HALS						
No/yes	2.920	1.140‐7.540	.026	0.180	0.637‐5.400	.257
Operation time						
<461.4 min/≥461.4 min	2.020	0.831‐4.920	.121			
Blood loss						
<350 g/≥350 g	0.923	0.391‐2.180	.855			
Blood transfusion						
No/yes	0.683	0.145‐3.210	.630			
Circular stapler size						
21 mm/25 mm	0.509	0.165‐1.570	.240			
DGC						
No/yes	5.600	2.160‐14.50	<.001	4.840	1.770‐13.30	<.001
EGC						
<650 mm^2^/≥650 mm^2^	1.340	0.563‐3.170	.511			

Abbreviations: BMI, body mass index; CI, confidence interval; DGC, distension of the gastric conduit; EGC, extension of the gastric conduit; HALS, hand‐assisted laparoscopic surgery; NAC, neoadjuvant chemotherapy; OR, odds ratio; TSE, thoracoscopic esophagectomy.

### 3.6 Body weight change and DGC

**FIGURE 5:**
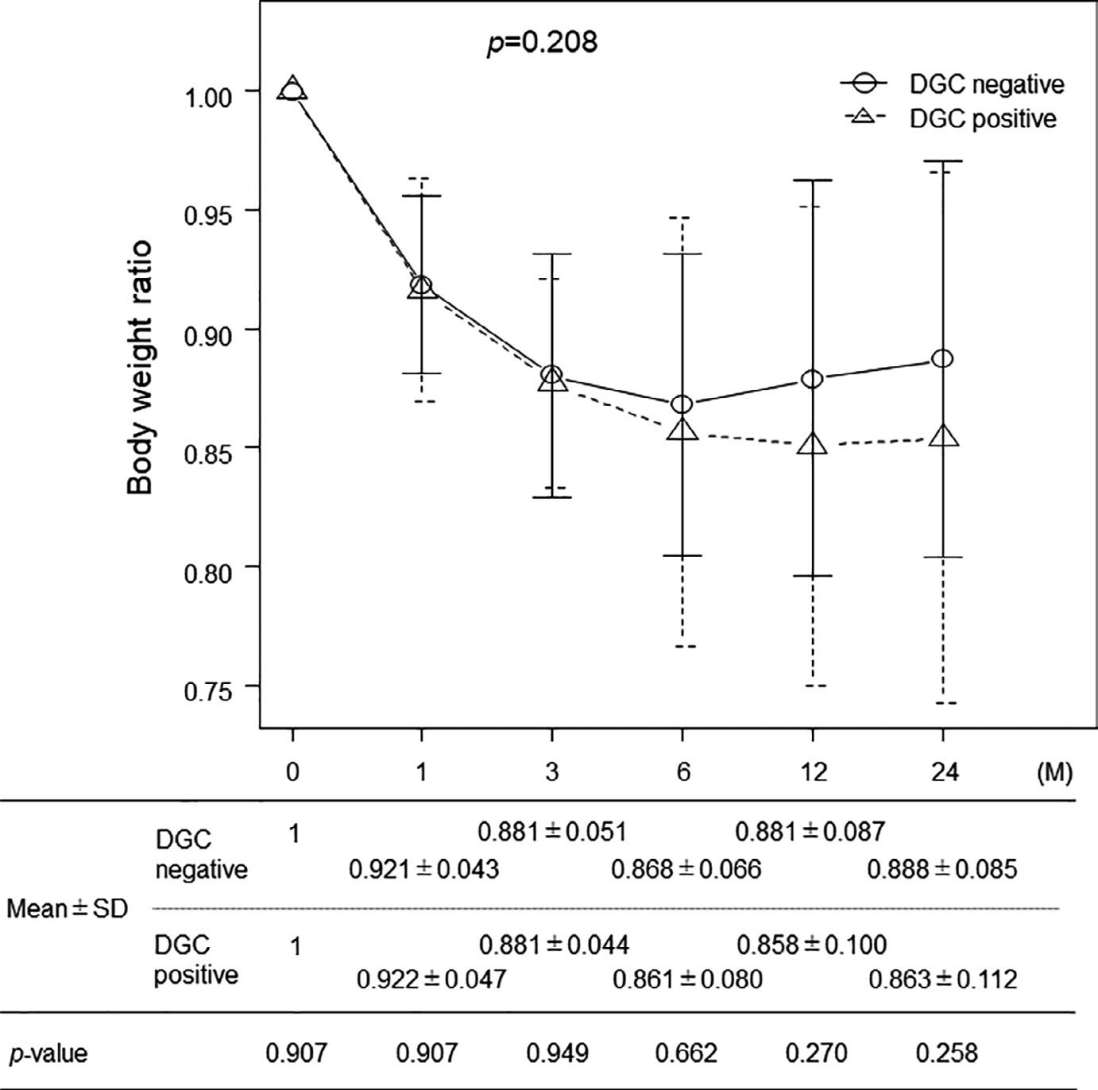
Relationship between DGC and postoperative body weight change. The body weight recovery of the DGC‐negative group tends to be better than that of the DGC‐positive group, although this intergroup difference was not significant. DGC, dislocation of the gastric conduit; SD, standard deviation

Among the 149 eligible patients, the patients who survived more than 2 years without recurrence were selected. As a result, the postoperative body weight change of 101 patients was analyzed (58 in the DGC‐negative group and 43 in the DGC‐positive group). The body weight recovery tended to be better in the DGC‐negative group than in the DGC‐positive group; however, this intergroup difference was not significant (Figure 5).

## 4 DISCUSSION

The present study is the first to demonstrate that DGC is an independent risk factor for anastomotic leakage and is closely related to other anastomotic disorders.

Anastomotic leakage is one of the most frequent and life‐threatening complications that often results in mediastinitis, mediastinal abscess, and empyema, especially in patients who undergo PM reconstruction.^13^ To improve peri‐ and postoperative QOL, it is very important to investigate the cause of anastomotic leakage and identify preventative measures. Although previous studies have reported that the causes of anastomotic leakage are tension, compression, ischemia, hyperemia, and an unskilled anastomotic technique, we focused on DGC because we had subjectively noticed that DGC was common in cases with anastomotic leakage.

Even in cases without anastomotic leakage, the gastric conduit is sometimes dislocated to the right pleural cavity after reconstruction via the PM route. Such dislocation may occur because of the weight of the gastric conduit itself or a mismatch between the volume of the gastric conduit and the mediastinal space after esophagectomy. The gastric conduit may be increasingly pulled toward the pleural cavity and distended by the negative breathing pressure. As seen in the present study, the dislocated gastric conduits became distended. However, distension of the gastric conduit was not found in the patients without DGC. Therefore, gastric distension must be partly a result of DGC. In many cases, the extension of gastric conduit was transient. It might be optimized according to the recovery of gastric motility from postoperative disorder.

The anastomosis was under strong tension, which may have caused the anastomotic leakage. The tension pulls the anastomotic site into the mediastinum, and pus pools in the mediastinum or pleural cavity. This phenomenon was more common in patients with a high BMI because of the large weight and volume of the gastric conduit due to the fatty greater omentum and the small mediastinal space due to the thick mediastinal fat tissue. Anastomotic leakage also leads to anastomotic stricture.

We often experienced the phenomenon that we could not pull the gastric conduit up to the level that we estimated in advance, and we could not avoid anastomosing at a more distal site than that we had planned. In such cases, we observed a finding suggestive of DGC (bending nasogastric tube to the right side) on X‐ray images just after the operation. However, there were cases where the finding suggestive of DGC was first observed on postoperative day 1 or later. In the cases where DGC occurs earliest, DGC must be the first cause of anastomotic disorder because the anastomotic site is strongly tensioned and more distal and ischemic due to bending. However, among the cases of DGC that occur after postoperative day 1, a gastric conduit that is too long may be the cause of anastomotic disorder and DGC. The anastomotic site of a gastric conduit that is too long must be ischemic, and the too long gastric conduit may be gradually pulled into the right pleural cavity. Thus, even though DGC may not be a cause of anastomotic disorder in every case, there is a significant relationship between DGC and anastomotic disorder.

DGE is a well‐known postoperative morbidity of esophagectomy. The incidence of clinically relevant DGE is in the range of 10%‐20%.^23^ In our study, 16.8% of all patients received medication for DGE. Although it was not significant, patients with DGC tended to have DGE. DGE might be influenced by the bend of the gastric conduit or negative pressure of the pleural cavity. Moreover, DGE probably affected postoperative body weight. According to the examination of body weight change, body weight recovery tended to be poorer in the DGC‐positive group. DGC may indirectly impair body weight recovery via DGE. However, the body weight recovery in patients with high BMI was poorer than that in patients with low BMI (data not shown). There is a possibility that the preoperative BMI was related to the difference in body weight recovery between the DGC‐positive and ‐negative group because the DGC rate was higher in patients with high preoperative BMI.

As mentioned above, DGC certainly has some unfavorable effects on patients who have undergone esophagectomy. Therefore, the strategy to prevent DGC is important. We usually pull the gastric conduit down to the abdominal cavity after esophago‐gastric anastomosis. In fatty cases, we attenuate the greater omentum accompanying the gastric conduit. However, very often the gastric conduit will not stay in the mediastinal space after esophagectomy. Because a strong upward vertical force may lead to straightening of the gastric conduit and avoid dislocation, we examined the effect of tumor location and anastomotic level on DGC. However, both were found to be unrelated. Nevertheless, we might not be able to avoid DGC using only a strong upward vertical force.

Numerous studies have evaluated the peri‐ and postoperative QOL of patients who have undergone PM vs retrosternal reconstruction after esophagectomy.^8^, ^9^, ^12^, ^18^, ^24^, ^25^ However, the optimal reconstruction route remains controversial, and the variation between studies may be due to the interstudy differences in the outcomes being evaluated. Regarding the frequency of anastomotic leakage, many studies have shown the superiority of the PM route.^26^, ^27^, ^28^ In the present study, we evaluated the incidences of anastomotic leakage and related morbidities, especially mediastinal abscess and empyema. PM reconstruction is reportedly inferior to retrosternal reconstruction regarding the incidences of mediastinal abscess, empyema, and mediastinal fistula.^14^, ^29^ However, the retrosternal route is a newly formed space that is separate to the pleural cavity, so it is less affected by negative pressure in the pleural cavity and by DGC. Thus, retrosternal reconstruction may result in less tension at the anastomotic site than PM reconstruction. Particularly in patients with a high BMI, gastric conduit reconstruction via the retrosternal route should be considered to avoid severe anastomosis‐related morbidities.

The mediastinal pleura is preserved when transhiatal esophagectomy is performed, resulting in a low risk of DGC. However, classical transhiatal esophagectomy does not allow the surgeon to perform adequate mediastinal lymphadenectomy. Therefore, to keep the oncological radicality, advanced methods such as mediastinoscope‐assisted transhiatal esophagectomy are required.^30^

The present study had some limitations. First, these operations were performed in a single institution. In such a situation, there is some possibility of continuing inadequate procedures. Second, this was a retrospective study, and the patients may have had various comorbidities that affected the incidences of postoperative morbidities. The present findings require confirmation in a multicenter prospective study that includes patients who undergo retrosternal reconstruction. Further studies will aid in the identification of the best reconstruction method in consideration of not only short‐term morbidity but also long‐term QOL.

## 5 CONCLUSIONS

DGC after reconstruction via the PM route is a significant cause of critical morbidities related to anastomosis. In particular, care is required when performing PM reconstruction in patients with a high BMI.

## DISCLOSURE

Conflict of interest: Masanobu Nakajima and all co‐authors declare no conflicts of interest for this article.

Ethical approval: The protocol for this research project has been approved by a suitably constituted Ethics Committee of the institution and it conforms to the provisions of the Declaration of Helsinki. Medical Ethics Committee of Dokkyo Medical University, approval number R‐20‐7J. All informed consent was obtained from the subjects.
